# Disordered eating, oral health and sports

**DOI:** 10.1038/s41415-026-9612-z

**Published:** 2026-02-27

**Authors:** Rebecca Moazzez, Deborah Bomfim, Renee McGregor, Rupert Austin

**Affiliations:** 093714340900597608818 Preventive and Restorative Dentistry Department, Arthur A. Dugoni School of Dentistry, University of the Pacific, USA; 560839344622447095620 Royal National Ear Nose Throat and Eastman Dental Hospitals, and University College London NHS Foundation Trust, London, United Kingdom; 684494353731163356536 Sports, Exercise and Medicine Dietitian, Clinical Director Team, United Kingdom; 391993165977593450144 Centre for Oral, Clinical and Translational Sciences Faculty of Dentistry, Oral and Craniofacial Sciences, King´s College London, United Kingdom

## Abstract

Multiple studies have identified that eating disorders are more common in elite athletes than in non-athlete populations. There are a wide range of types of eating disorders that can impact the oral and general health of athletes, including anorexia, bulimia, binge eating disorder, avoidant restrictive food intake disorder and other specified feeding and eating disorder. In addition, athletes can be affected by disordered eating such as relative energy deficiency in sport. This paper will explore what we know about the impact of eating disorders on oral health of athletes. We will consider suggested approaches to conversations with those at risk of an eating disorder and consider sources of support for affected individuals. This narrative paper is targeted at all providers of oral healthcare and oral health policy, as well as athletes and sports medicine practitioners and allied professionals.

## Key points

- Eating disorders are common in elite athletes, especially those participating in aesthetic sports that emphasise body appearance. Many athletes will be at increased risk of erosive tooth wear and other oral conditions as a result.
- Elite athletes may feel able to open up to dental care professionals regarding an eating disorder and therefore it is important for dental care professionals to be aware of the common signs and symptoms of an eating disorder.
- Prevention is key to avoid the long-term sequalae that can result if erosive tooth wear and other oral conditions progress.

## Introduction

Eating disorders are complex and serious mental illnesses which can be difficult to diagnose and manage. Early diagnosis facilitates effective management of an eating disorder, whereas conversely, the more embedded the eating disorder is, the more difficult it is to manage. Eating disorders are inherently more likely to develop in athletes due to the very nature of elite sports, where the importance of body composition and aesthetics is of paramount importance to performance.^1^ The prevalence of eating disorders has been shown to be greater in elite athletes than the general public.^2^ Elite athletes are especially at risk of disordered eating; studies have shown that up to 84% of college athletes at some time engaged in disordered eating or weight control behaviours.^3^

The oral health implications of an eating disorder are widespread and deleterious in many respects to good oral and general health. These risks include severe generalised erosive tooth wear, especially if the eating disorder has been ongoing for a long time.^4^ Therefore, general dental practitioners and sports dentists are uniquely placed to help identify oral signs and provide athletes with support in getting help. The wider dental community has significant potential to help spread the message to the wider sports community about the effects of eating disorders on oral health in order to help prevent disordered eating impacting the oral health of athletes and the wider population.

While elite sport and disordered eating have clear links, there is relatively little known about how dentists can promote good oral health among this group of patients. This is potentially a missed opportunity, as dental care professionals may be among the first healthcare professionals to spot early signs and symptoms of an eating disorder, when it has the best chance of being successfully treated. In the early phase of disordered eating, there is the best chance of preventing the disease causing lifelong deleterious ramifications.

As sport dentists, there is a significant opportunity to pick up the signs and symptoms as the prevalence of eating disorders is higher among elite athletes than the general population.^5^ However, since eating disorders can have serious physical and mental health consequences, their management must be considered multidisciplinary with recommendation for referral to a medical practitioner. Depending on the severity of the eating disorder, there may also be safeguarding issues for the safety of the patient to consider for the dental practitioner. This paper will review disordered eating before considering the current state of knowledge among the dental profession regarding disordered eating and oral health in elite sport. The paper will outline the opportunities for sports dentists in managing eating disorders and promote the importance of early diagnosis to help mitigate oral and general health complications and identify patients for onward referral.

## Overview of eating disorders in athletes

Eating disorders are biologically based serious mental illnesses which, although fully treatable with a combination of nutritional, medical and therapeutic support, can be chronic problems with high risk of relapse.^6^^,^^7^ Early identification and diagnosis are paramount. The sooner someone gets the treatment they need, the better the chance of a good recovery. It is essential to recognise that eating disorders are not choices, passing fads or phases. Eating disorders are severe and can be fatal, through either suicide or as a result of the sequalae that occur if untreated. Box 1 shows the long-term consequences if an athlete is not successfully treated for disordered eating behaviours. These consequences can take time to develop as the cardinal signs of an eating disorder can be recognised by a persistent pattern of behaviours that can cause health problems and/or emotional and social distress.

Thus, for affected individuals, feeding and eating disorders are characterised by a persistent disturbance of eating or eating-related behaviour. These behaviours result in altered consumption or absorption of food which then significantly impairs physical health or psychosocial functioning. A key understanding is that these behaviours are often initially used as a method of avoiding emotional risk and are therefore perceived as a coping strategy or a ‘helpful' behaviour. However, with time and as the behaviours become more embedded, they then become a learned behaviour. These learned behaviours provide a false sense of security and thus are difficult for the individual to let go of without appropriate intervention and support.

Eating disorders cover a wide range of dysfunctional relationships with food and it is important to be aware that people with eating disorders do not have a particular look. However, there may be some signs and symptoms to look out for that may suggest that an individual is struggling with eating issues.

Eating disorders are usually diagnosed by strict criteria by a specialist medical professional. Regardless of which type of eating disorder is affecting an athlete, they all involve obsessive thoughts about eating food and body image, most of the time that severely impacts quality of life. They can be combined with excessive exercise or exercise dependency in athletes. The main categories of clinical eating disorders are:

- Anorexia nervosa
- Bulimia nervosa
- Binge eating disorder
- Avoidant restrictive food intake disorder (ARFID)
- Other specified feeding and eating disorder.

Box 1 Long-term consequences if an athlete is not successfully treated for disordered eating behaviours

- Metabolic imbalances
- Increased risk of athletic injury
- Poor sports performance
- Inability to perform sports requirements resulting in quitting/retirement
- Decreased training response
- Impaired judgement
- Decreased coordination
- Impaired aerobic functioning
- Damage to vital organs
- Loss of menstrual cycle/infertility issues
- Increased risk of heart failure and cardiovascular complications
- Bone and muscle loss
- Ulcers
- Gastrointestinal complications
- Caries/periodontal disease/erosive tooth wear/loss of teeth
- Increased depression/anxiety
- Increased risk of substance abuse
- Increased suicidal thoughts
- Death

### Anorexia nervosa in athletes

Anorexia nervosa is characterised by self-starvation and weight loss resulting in low weight for height and age. Anorexia has the highest mortality of any psychiatric diagnosis.^8^ Body mass index – a measure of weight for height – is typically under 18.5 in an adult individual with anorexia nervosa. Box 2 lists signs of disordered eating and exercising that may point to anorexia nervosa. Athletes who engage in aesthetic sports that emphasise body appearance are particularly at risk for engaging in these behaviours.^9^

Box 2 Signs of disordered eating and exercising that may point to anorexia nervosa^6^

- Fixation on body weight, shape or size
- Calorie counting
- Intense fear of gaining weight
- Preoccupation on contents and nutritional aspects of food
- Declining/skipping meals
- Refusing to eat in front of others
- Food rules
- Distorted body image
- Participation in an aesthetic sport (dance, gymnastics, figure skating, wrestling, equestrianism)
- Unusual food behaviours
- Reported lethargy, difficulty with stamina
- Impaired concentration

### Bulimia nervosa in athletes

Individuals with bulimia nervosa typically alternate dieting or eating only low-calorie ‘safe foods' with binge eating on ‘forbidden' high-calorie foods. Binge eating is defined as eating a large amount of food in a short period of time associated with a sense of loss of control over what, or how much one is eating. Binges occur at least weekly and are typically followed by what are called ‘compensatory behaviours' to prevent weight gain. These can include fasting, vomiting, laxative misuse, or compulsive exercise. Individuals with bulimia nervosa can be slightly underweight, normal weight, overweight or obese. Box 3 outlines the signs which may signify an athlete is engaging in bulimic behaviours.^6^ These behaviours are more common in athletes than anorexia nervosa as athletes require increased nourishment to fuel their activities.

Box 3 Signs which may signify an athlete is engaging in bulimic behaviours^6^

- Binge eating behaviours after practice, interrupted or followed by trips to the bathroom
- Eating in secret
- Hiding food
- Preoccupation with body weight/shape/size
- Distorted body image
- Eating beyond fullness
- Expressing shame or guilt around eating
- Possessing/purchasing diuretics and/laxatives
- Excessive coffee drinking/fluid consumption
- Scarring on the knuckles

### Binge eating disorder in athletes

As with bulimia nervosa, people with binge eating disorder have episodes of binge eating in which they consume large quantities of food in a brief period, experience a sense of loss of control over their eating and are distressed by the binge behaviour. Unlike people with bulimia nervosa, however, they do not regularly use compensatory behaviours to get rid of the food by inducing vomiting, fasting, exercising, or laxative misuse. Binge eating disorder can lead to serious health complications, including obesity, diabetes, hypertension, and cardiovascular diseases. Box 4 lists signs of binge eating in athletes, which are linked to engaging in restrictive behaviours or appearing in public to not meet nutritional needs according to their energy output.

Box 4 Signs of binge eating in athletes

- Eating in secret
- Hiding food
- Expressing shame/guilt around food and/or eating
- Eating at a fast pace
- Reported feelings of depression or low self-worth
- Nourishment in public is observed to be inappropriate with the amount of energy output
- Weight fluctuations
- Lethargy

### Avoidant restrictive food intake disorder

ARFID is a recently defined eating disorder that involves a disturbance in eating resulting in persistent failure to meet nutritional needs and extreme picky eating. The impact on physical and psychological health and degree of malnutrition can be like that seen in people with anorexia nervosa. However, people with ARFID do not have excessive concerns about their body weight or shape and the disorder is distinct from anorexia nervosa or bulimia nervosa.

### Other specified feeding and eating disorder

This diagnostic category includes eating disorders or disturbances of eating behaviour that cause distress and impair family, social or work function but do not fit the other categories listed here. In some cases, this is because the frequency of the behaviour does not meet the diagnostic threshold (e.g., the frequency of binges in bulimia or binge eating disorder) or the weight criteria for the diagnosis of anorexia nervosa are not met.

## Overview of disordered eating in athletes

### Disordered eating

Disordered eating involves the pre-occupation with irregular thoughts about food, eating and body image but that do not dominate each day. This may or may not warrant a formal diagnosis. There is usually an awareness and maybe pre-occupation with exercise and body image but not to the point that it becomes obsessive or all-consuming. This can still negatively impact quality of life. It often constitutes feelings of guilt and shame associated with eating, and there are often thoughts around ‘needing to earn' food, or following rules centred around eating, such as ‘eating clean'. If behaviours become too restrictive this can tip into a more serious eating disorder.

### Relative energy deficiency in sport

There is also a relatively new presentation, mainly associated with those who participate in physical activity and sports known as REDs (relative energy deficiency in sport) but can impact anyone who is physically active.^10^ This occurs when there is not enough energy in the body relative to the amount of energy that is being used through exercise and or movement. It is a multi-system condition that results in a physiological decline in the body that can result in poor physical health, mental health and performance outcomes. As highlighted in the 2023 International Olympic Committee's consensus statement on REDs, the condition is underpinned by low energy availability (LEA), where energy availability is the amount of energy available for biological function once the cost of movement has been subtracted from overall energy intake. When there is not sufficient energy available, it is known as LEA.

Collectively, LEA, insufficient recovery and holding your body at a body composition that is too low for requirements all generate stress within the body and it is this stress that, over time, results in the symptoms associated with REDs, which include multiple health sequelae, including reduced growth and development, sleep disturbances, mental health issues and impaired bone health.^10^ It seems reasonable to speculate that REDs might affect oral health in view of the impacts on immune function, bone health and mental health, among others. Therefore, awareness of REDs should raise dentists' insight into another potential impact of eating disorders on oral health, especially as a call for further research.

#### Types of REDs

- Intentional REDs, often associated with psychological involvement, with the individual presenting with an eating disorder, disordered eating and/or exercise dependency
- Unintentional REDs or accidental REDs occurs as a result of an unintentional energy imbalance or insufficient recovery, for example, this might occur during a period of heightened performance demands, or during a period of extensive travel where food and rest are difficult to maintain.

## Types of eating disorder with relevance to sports

The exact prevalence of eating disorders in sport is not known. It is assumed the percentage of athletes living with disordered eating is potentially up to 50% of the athletic population, as individuals undertake these behaviours in isolation.^11^

Indeed, numerous global studies comparing the athlete population with their non-athlete peers for age have concluded that there is a 20% higher prevalence of disordered eating in athletes.^12^ Research has also highlighted that individuals with indicated eating disorders are over 3.5 times more likely to also have exercise addiction.^13^ While this relates more to the recreational athlete, it still shows the strong links between dysfunctional relationships with food, exercise and fitness.

There are some personality types that have a higher susceptibility to developing an eating disorder. Genetically they may have a predisposition that makes them more vulnerable, especially when this personality is combined in certain psychosocial environments.^14^

The attributes we often see in athletes – high perfectionism, focus, determination, enthusiasm, highly critical – overlap significantly with personality traits commonly associated with eating disorders.^14^ These include high perfectionism, impulsivity, harm avoidance, reward dependence, sensation seeking, neuroticism, and obsessive-compulsiveness, in combination with low self-directedness, assertiveness, and cooperativeness.

Thus, the same personality traits that encourage optimal athletic performance, when placed in a highly competitive environment such as professional sport or fitness, may create the perfect storm for dysfunctional behaviours involving food, exercise and body image to occur.

## What do we know about disordered eating and oral health in elite sport?

Oral health is consistently poor for elite athletes especially considering the young age of this cohort of the population. Among athletes, prevalence of dental caries, periodontal diseases, dental erosion and pericoronitis may be higher than in the general population.^5^ With specific regards to the role of disordered eating, there is a strong association between the most common eating disorders of anorexia nervosa, bulimia nervosa and binge eating disorders and oral conditions, which is partly directly related to the nature of the eating disorder itself and secondarily related to a lack of proper nourishment and optimal body functioning.^2^

### Impact of eating disorders on oral and dental health

**Fig. 1:**
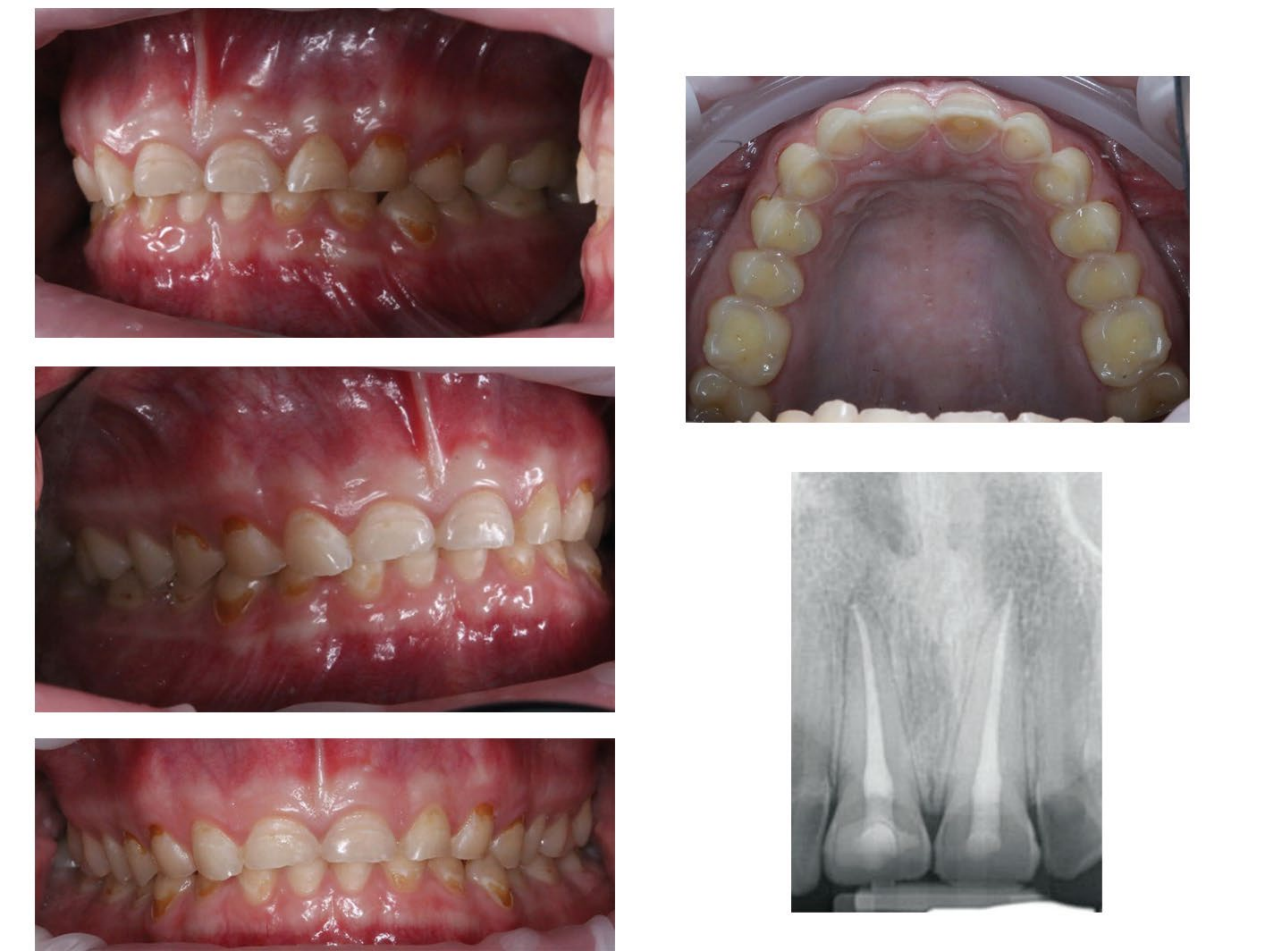
Clinical images of the oral effects of an eating disorder including erosive tooth wear, dental caries and endodontic sequalae

Box 5 outlines the potential oral health complications from eating disorders, which can be severe and lifelong if not diagnosed early.^5^^,^^15^ These oral health complications are exemplified by the clinical images shown in Figure 1 which show clinical images of the oral effects of an eating disorder including erosive tooth wear, dental caries and endodontic sequalae. The severe anterior maxillary erosive tooth wear, which is rapidly progressing and affecting the palatal and lingual surfaces of the teeth is characteristic of that caused by eating disorders, especially in athletes.^16^ The erosive tooth wear has led to exposure of the dental pulps of the maxillary central incisors and consequent endodontic pathology. Unusually, the erosive tooth wear is also accompanied by bucco-cervical carious lesions, which is a presentation of caries more commonly seen in post-radiation caries or other high-caries risk groups.

Box 5 Potential oral health complications from eating disorders

- Erosive tooth wear
- Dental caries
- Burning tongue
- Angular cheilitis
- Dry lips
- Atrophic mucosa
- Increased risk of periodontal diseases
- Oral ulcerations
- Taste impairment
- Candidiasis
- Parotid gland enlargement
- Altered salivary flow rate

## What are the opportunities for sports dentists in managing eating disorders in athletes?

Sports dentists may be the first clinicians to observe the health consequences of an eating disorder since symptoms arising from erosive tooth wear may lead an athlete to seek professional help. Furthermore, as dental care professionals (including dental hygienists and dental therapists) are often outside of athletes' medical care, they may be viewed as a ‘safe space' for an athlete to open up about an eating disorder.^17^ Consequently there have been concerted efforts to raise awareness of the importance of good oral health among athletes.^2^^,^^5^ Patients with a suspected eating disorder should be advised that they are in need of referral to a medical practitioner and if consented, the referral should be made promptly. As with other psychological issues presenting in dental patients, the dental professional may need to consider safeguarding issues if the disorder appears a serious threat to their physical and mental health. Local safeguarding protocols and support should be urgently consulted.^18^ There are useful strategies to encourage patients to engage with dental care, including avoiding attempting to force a particular course of action on patients. Useful ways to engage are listed below:

- Communicating concern and empathy
- Remaining calm
- Avoiding letting common myths cloud your perceptions
- Showing that you understand that there may be something other than food or weight troubling the person
- Understanding an eating disorder as a coping mechanism should inform your initial approach
- Asking questions about the duration and severity of the disorder such as the frequency of binging or purging, and any current medical treatment the patient is engaged with
- Not focusing on specific behaviours any longer than you need; move the focus from specific behaviours to asking how the person is feeling or whether there is something going on for them they would like to talk about
- Avoiding criticism or suggesting quick fixes.

Ultimately, the aim is to encourage the patient to allow referral to other health professionals such as a general practitioner, dietitian, psychiatrist, psychologist or other specialist. For those aged younger than 18, it may be advisable to raise any concerns with the patient's parent(s)/guardian(s) and refer to a clinician for expert opinion and support. Finally, building a therapeutic relationship should encourage the patient to return regularly for dental care.

There are many resources available to help dental professionals to engage with patients with eating disorders, including those that provide suggested key phrases and questions to use during a dental consultation with a patient with eating disorders:^19^

- ‘I am noticing _____________on your gums/teeth/throat/tongue'
- ‘This is something I have seen with people who purge food by vomiting/_____________'
- ‘Can you tell me a little more about the behaviours that might be having this effect on your mouth/teeth/gums/throat?'
- ‘I am concerned for you, and it seems that there is an issue here that needs to be addressed'
- ‘Do you have help available to you at the moment?'
- ‘Is there someone you might feel comfortable talking to?'
- ‘Because an eating disorder can affect your oral, mental and physical health, it is very important to seek professional advice and some additional support'
- ‘While you are seeking help, I would like to suggest some immediate options for improving your oral health'.

## Conclusion

Eating disorders are a complex range of severe mental health conditions that present more commonly in athletes than other patient groups. Dental care professionals have a key role in early identification of the signs and symptoms of eating disorders in athletes and in arranging referral to medical practitioners when appropriate. It is important for dental professionals to build therapeutic relationships with affected individuals with an aim of helping patients to focus on their long-term health and wellbeing beyond their sporting career as without treatment, there can be lifelong negative consequences from the eating disorder.
